# Polycystin-1 Enhances Stemmness Potential of Umbilical Cord Blood-Derived Mesenchymal Stem Cells

**DOI:** 10.3390/ijms22094868

**Published:** 2021-05-04

**Authors:** Se-Hwa Jung, Ji-Eun You, Soon-Won Choi, Kyung-Sun Kang, Je-Yeol Cho, Jungmook Lyu, Pyung-Hwan Kim

**Affiliations:** 1 Department of Biomedical Laboratory Science, Konyang University, Daejeon 35365, Korea; fish1206@naver.com (S.-H.J.); jean9643@naver.com (J.-E.Y.); 2 Adult Stem Cell Research Center and Research Institute for Veterinary Science, College of Veterinary Medicine, Seoul National University, Seoul 08826, Korea; nomnoos@gmail.com (S.-W.C.); kangpub@snu.ac.kr (K.-S.K.); 3 Department of Biochemistry, College of Veterinary Medicine, Seoul National University, Seoul 151-742, Korea; jeycho@snu.ac.kr; 4 Myung-Gok Eye Research Institute, Department of Medical Science, Konyang University, Daejeon 320-832, Korea; ujm@konyang.ac.kr

**Keywords:** *PKD1*, umbilical cord blood-derived mesenchymal stem cell (UCB-MSC), stemness, osteogenic differentiation

## Abstract

Polycystic Kidney Disease (*PKD*) is a disorder that affects the kidneys and other organs, and its major forms are encoded by polycystin-1 (*PC1*) and polycystin-2 (*PC2*), as *PKD1* and *PKD2.* It is located sandwiched inside and outside cell membranes and interacts with other cells. This protein is most active in kidney cells before birth, and *PC1* and *PC2* work together to help regulate cell proliferation, cell migration, and interactions with other cells. The molecular relationship and the function between *PKD1* and cancer is well known, such as increased or decreased cell proliferation and promoting or suppressing cell migration depending on the cancer cell type specifically. However, its function in stem cells has not been revealed. Therefore, in this study, we investigated the biological function of *PC1* and umbilical cord blood-derived mesenchymal stem cell (UCB-MSC). Furthermore, we assessed how it affects cell migration, proliferation, and the viability of cells when expressed in the *PKD1* gene. In addition, we confirmed in an ex vivo artificial tooth model generated by the three-dimension printing technique that the ability to differentiate into osteocytes improved according to the expression level of the stemness markers when *PKD1* was expressed. This study is the first report to examine the biological function of *PKD1* in UCB-MSC. This gene may be capable of enhancing differentiation ability and maintaining long-term stemness for the therapeutic use of stem cells.

## 1. Introduction

Cell therapy is one of the basic treatments used in regenerative medicine and tissue engineering. Among the cells used in cell therapy, stem cells are now used as an indispensable element in the development of artificial organs called organoids. Stem cells are an undifferentiated state cell that has self-renewal ability and is a pluripotent cell that can develop into any tissue [1]. It is also known to have various functions such as peripheral secretion signal, immune regulation, apoptosis prevention, and blood supply by angiogenesis [2]. There are many reports that stem cell-based cell therapies have excellent effects and application ability, and various studies are under-explored for the clinical use of stem cells [3].

However, stem cells still have a long way to go due to the major shortcomings, such as the limitations of cell viability, cell proliferation, time-dependent cell passage, cell expansion, and the differentiation into desired cells, in an administered site in vivo. Its curative efficacy will be significantly greater than it is now if they can overcome the disadvantages in stem cell-based cell therapies. Many studies have introduced various methods to overcome the shortcomings of stem cells. It has been researched to improve the viability and differentiation of stem cells by enhancing the function of stem cells through genetic engineering and producing stem cell complexes using biocompatible materials and nanomaterials. New and fusion methods are required to further enhance the efficacy of stem cells [2,3,4]. From this point of view, we focused on PKD genes as genetic material to overcome the obstacles of stem cells.

Polycystic kidney disease (*PKD*) is a genetic disorder in which a specific protein is generated by an abnormal gene and the kidney tubules are abnormally deformed, causing multiple cysts in the kidney to cause developmental disorders [5,6,7,8]. When this protein causes multiple cysts filled with fluid in the nephron in the kidney, the size of the kidney becomes larger than normal, and the tissue is damaged, leading to physiological dysfunction [9].

Eighty-five percent of *PKDs* are caused by mutations in the polycysin-1 protein of the gene. Polycystin-1 is called *PKD1* or *PC1*, and this protein is the most active in kidney cells before birth and interacts and functions with polycystin-2 *(PKD2*) [10]. According to the current study, there is no precise treatment with the development of *PKD* caused by mutation, and the correlation with other cells has not been studied much [11,12,13,14]. Only a study on the correlation with cancer cells was recently published. Although it is known that *PKD1* affects cell growth, proliferation, and migration, the results were observed differently depending on cancer cell lines [15]. *PKD1* also regulates cell proliferation and cell migration through mammalian target of rapamycin (mTOR) and Janus kinase (JAK) signals, but in the lung cancer cell line (A549), *PKD1* inhibits cell migration and functions as a tumor-suppressor protein in some cases. On the other hand, the glioblastoma cell line (GOS3) showed different results in the cell line, such as acting as a tumor gene protein by improving cell migration, but the exact mechanism has not been revealed [12,15,16]. There is still a controversial part about the function of the *PKD* gene, but it affects cell growth, proliferation, and migration with *PKD2* [7,10,17,18]. Although there are various reports of *PKD* gene in cancer cell, the function and relationship between the *PKD* gene and stem cells has not been reported so far.

Therefore, in this study, we investigated whether the *PKD1* gene as genetic material can overcome its shortcomings and its biological function in stem cells.

## 2. Results

### 2.1. Expression of PKD1 at Transcript and Protein Level in Various Cell Lines

**Figure 1:**
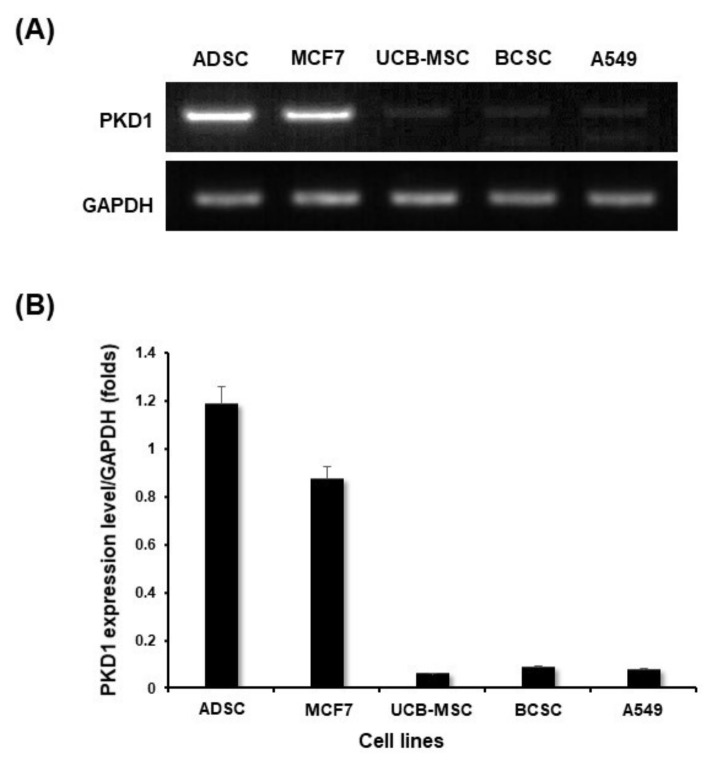
Comparison of PKD expression level according to each cell line. (**A**) The expression level of the *PKD1* gene was confirmed by RT-PCR using mRNA extracted from each cell in the transcript level; (**B**) Quantitative values of *PKD1* expression were normalized by GAPDH.

**Figure 2:**
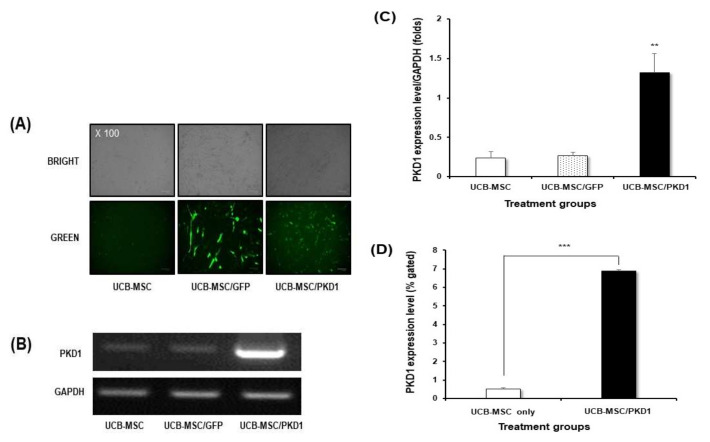
Validation of *PKD1* expression in transcript and protein level by *PKD1*-expressing GFP/lentiviral vector. (**A**) GFP expression in UCB-MSC transfected with *PKD1*-expressing vector. *PKD1* expression (**B**) and quantitative data (**C**) in transcript level of *PKD1* expressing UCB-MSC; (**D**) The expression of *PKD1* at the protein level. Data shown represent the mean ± SD (*n* = 3). ** *p* < 0.02 or *** *p* < 0.01 versus UCB-MSC only group.

In order to confirm the expression level of PKD1 in stem cells, RNA at the transcriptome level was extracted from various cell lines, and then cDNA was synthesized by RT-PCR from each cells. The various cells used for this experiment are adipose-derived mesenchymal stem cell (ADSC), breast cancer cell MCF7, MCF7 breast cancer-derived stem cell (BCSC), lung cancer cell A549, and umbilical cord blood-derived mesenchymal stem cell (UCB-MSC). After conventional PCR for the detection of PKD1 gene, GAPDH was normalized and graphed to quantify the data [19]. As shown in the results of Figure 1, the expression level of the PKD1 gene was differently observed by cell lines. Among them, PKD1 expression in UCB-MSC and BCSC was observed lower compared with those of other cells. Especially, stem cells showed different PKD1 expression depending on their derived origins. As shown in Figure 1A, quantitative data of the detected band of PKD1 also showed a similar tendency (Figure 1B). Therefore, based on this result, we generated over-expression vectors tagged with the GFP gene to identify the function of PKD1 in this stem cell. Lentiviral vector with PKD1 and GFP expression cassette was characterized and validated in Figure S1. The expression of PKD1 in UCB-MSC by this vector was confirmed in the results of Figure 2. Green fluorescent protein-expressing vector (gWIZ/GFP) was used as positive control against *PKD1*-expressing vector.

First, transfection by each vector was confirmed via the expression of GFP (Figure 2A). Although there is a difference in the levels of GFP expression according to the characteristics of the vector, *PKD1*-expressing vector was well transfected despite stem cells being known to have poor transfection efficiency. For this, *PKD1* vector with a large gene size of 12 Kbs was transfected twice to induce an efficient expression of gene under the conditions of minimal toxicity. Next, we checked at the transcript level through RNA extraction after transfection to confirm whether the *PKD1* gene is well expressed by the transfected *PKD1* vector. As shown in the result in Figure 2B, the highest *PKD1* expression was examined in the cells transfected with *PKD1* vector. The transfected *PKD1* vector induced higher *PKD1* expression than those of other groups. These patterns were similarly observed in quantitative data (Figure 2C).

*PKD1* spans the cell membrane of cells, with one end of the protein present in the cell and the other protruding out of the cell for the extracellular mechanical stimulation into intracellular biochemical signals [15,16,17,18]. Therefore, we examined whether *PKD1* is actually well expressed at the protein level by *PKD1* expression vector via flow cytometry. As shown in the flow cytometer analysis (Figure 2D), the cell transfected with *PKD1* vector showed the increased *PKD1* expression compared with that of non-transfected cells. Taken together, these results mean that the *PKD1* expression system works well in UCB-MSC.

### 2.2. The Biological Functions of PKD1 in UCB-MSC Effects of PKD1 on Cell Expansion and under the Condition of Oxidative Stress in UCB-MSC

**Figure 3:**
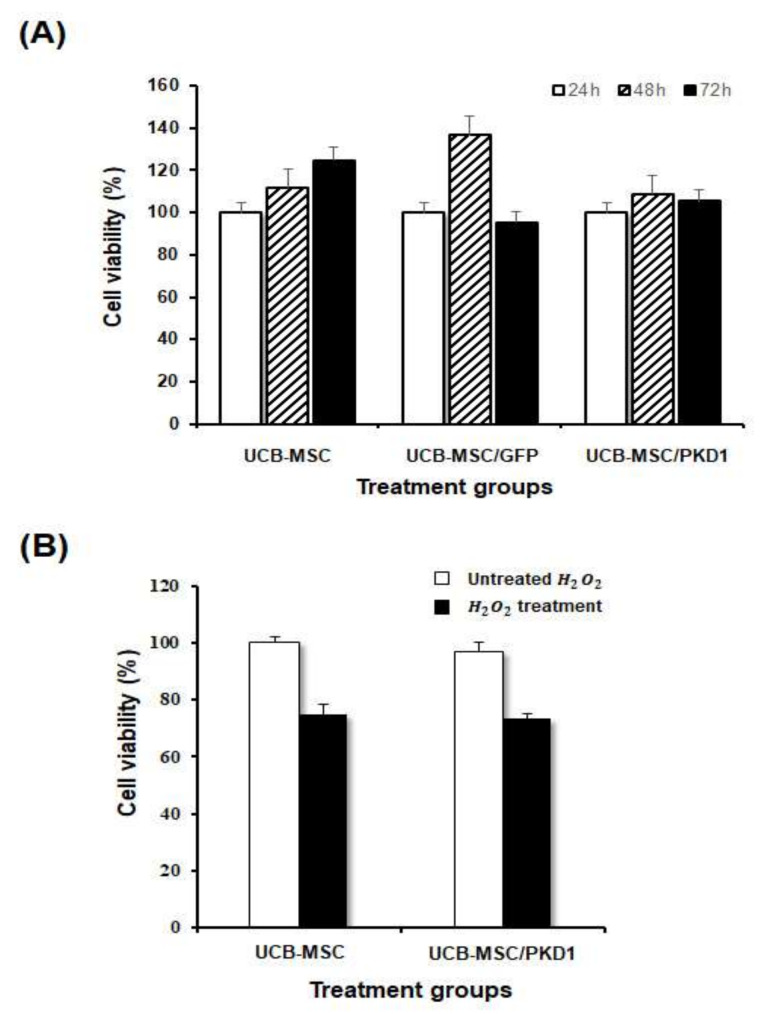
Biological effects of *PKD1* in UCB-MSC. To assess the biological function of *PKD1* in *PKD1*-expressing UCB-MSC, *PKD1* vector was transfected, and then after 48 h, cell proliferation and antioxidant effect were examined. (**A**) Cell proliferation in a cultured time-dependent manner by MTT assay; (**B**) The resistance effect against oxidative stress induced by H_2_O_2_. Data shown represent the mean ± SD (*n* = 3).

After checking *PKD1* by *PKD1*-expressing vector, we assessed how this gene actually affects in stem cells when it is expressed. It is known that *PKD1* has a function of cell proliferation, but some studies now report that the function of *PKD* varies from cell to cell. To examine this in stem cells, a cell proliferation experiment was carried out by MTT assay in a time-dependent manner. In the result of Figure 3A, cell viability did not increase significantly in *PKD1*-transfected UCB-MSC compared with those of other treated groups at all the times tested. This result demonstrates that the expression of *PKD1* did not affect cell proliferation in UCB-MSC, although *PKD1* originally had a function of cell proliferation.

It is known that the *PKD* gene correlates with the oxidative stress pathway in relation to the RTK/MAPK pathway in other cancer cell lines, including bladder cancer [20,21,22]. Therefore, we evaluated whether *PKD1* expressed in stem cells is resistant to oxidative stress. After selecting the optimal concentration of hydrogen peroxide (H_2_O_2_) to mimic oxidative cellular damage, the antioxidant function of *PKD* was evaluated.

As a result, H_2_O_2_ well induced the cytotoxicity in UCB-MSC and the cell viability of UCB-MSC transfected with *PKD1* also decreased (Figure 3B), which is consistent with the above proliferation result (Figure 3A). In addition, there was no significant difference between the results of *PKD1*-expressing cells treated with H_2_O_2_ (UCB-MSC/*PKD1* + H_2_O_2_ group) and non-transfected cells treated with H_2_O_2_ (UCB-MSC + H_2_O_2_ group). This result means that the *PKD1* gene is ineffective against oxidative stress.

**Figure 4:**
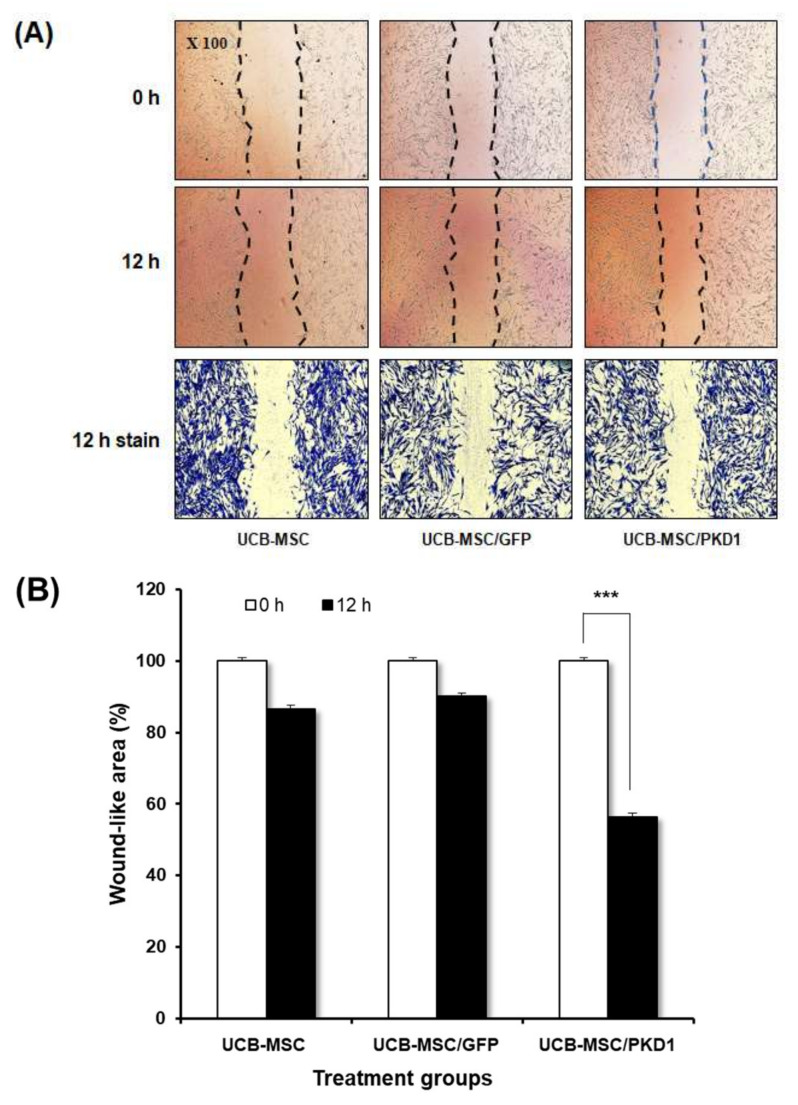
Improved cell migration ability of UCB-MSC by *PKD1* expression. (**A**) To the migration ability of UCB-MSC in the presence of *PKD1* expression, the cells were scratched by a 200 uL pipette tip. At 12 h, the cells were stained with crystal violet; (**B**) Open area was calculated using image J and quantified by percentage values. Data shown represent the mean ± SD (*n* = 3). *** p < 0.01 for the comparison of 0 h and 12 h in *PKD1* expressing UCB-MSC.

*Polycystin-1* also regulates cell proliferation and cell migration through mTOR and JAK signals in the cancer cell line [15,17]. Based on this fact, we evaluated whether *PKD1* gene affects the migration of stem cells. For this, when the cell confluence reached 80%, scratches were given at regular intervals with a 200 μL pipette tip, and the same part was brightly photographed at 0 and 12 h using a microscope. At 12 h, the cell was stained with crystal violet solution to more accurately check the degree of migration (Figure 4A). The scratch area at 0 h and the scratch area at 12 h were photographed with a microscope and then measured and graphed to confirm the reduced area using image J (Figure 4B). Using Pixels as the mean value, the pixels at 0 and 12 h were measured by repeating three times each, and the average values for each group measured by image J are shown in the Table S1. As shown in the results, all groups showed a decreased area at 12 h compared to 0 h. However, UCB-MSC transfected with *PKD1* significantly observed a decreased area by migrated cells at 12 h compared with that of 0 h. These results demonstrate that *PKD1* has a great influence on the migration of UCB-MSC, meaning that this could improve stem cell mobility to target areas.

### 2.3. The Improved Differentiation Ability of Stem Cells by PKD1

Stem cells have a stem cell ability called stemness, which not only improves the ability of cells to self-renew but also transforms into various tissues such as organs and bones. Enhancement of stemness may regenerate the damaged human body and improve the ability to treat diseases. From this point of view, we evaluated whether *PKD1* can function effectively in enhancing the stemness of UCB-MSC. For this, three kinds of representative stemness biomarkers, Oct4 (octamer-binding transcription factor 4), Nanog, and Sox2 (SRY-Box Transcription Factor 2), were evaluated through RT-PCR [23].

**Figure 5:**
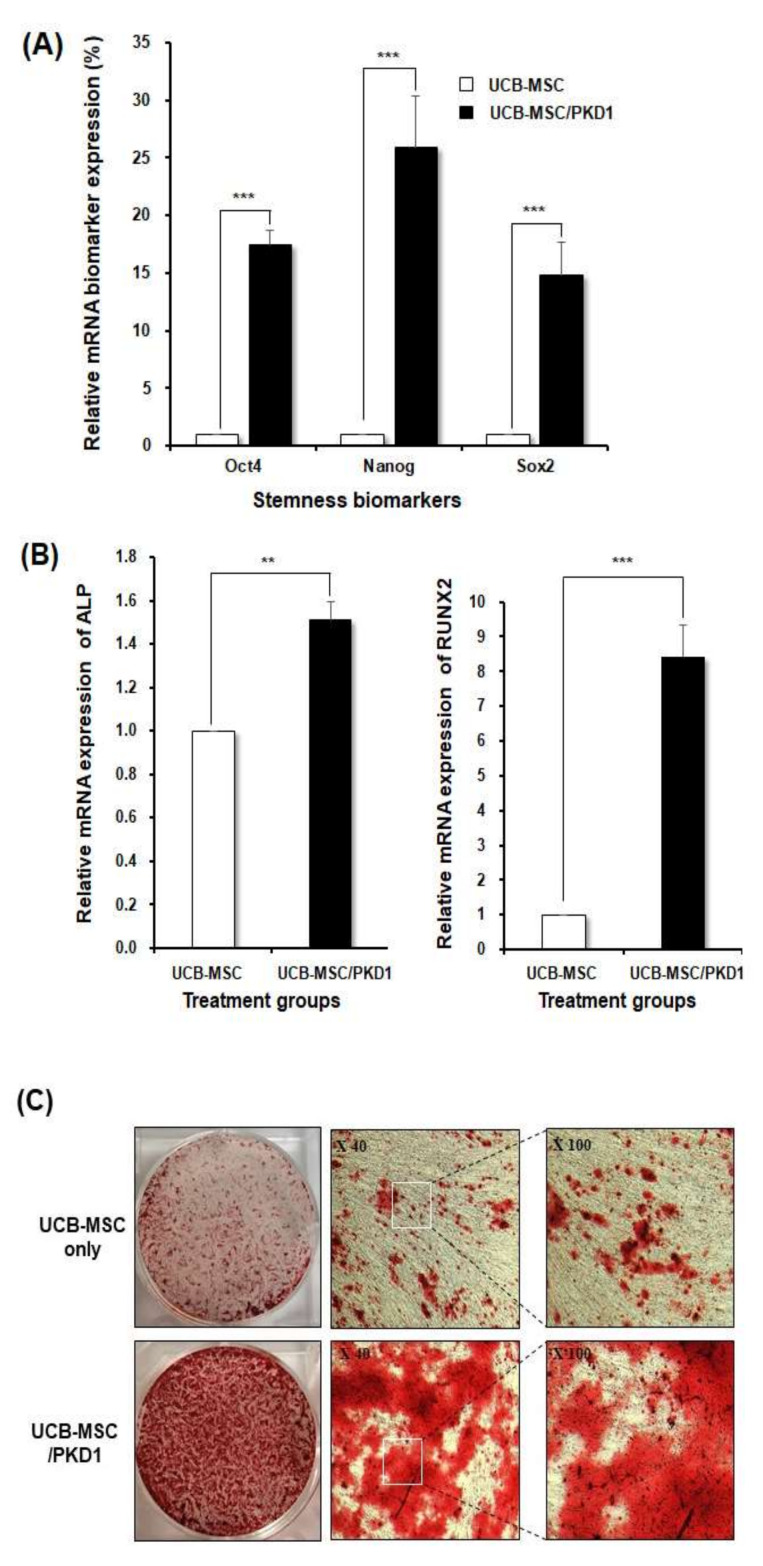
The expression of the increased stemness biomarkers and improved osteogenic differentiation ability of *PKD1*-expressing UCB-MSC. To evaluate the potential of stem cells by *PKD1* transfection, the expression of stemness markers Oct4, Nanog, and Sox2 was detected by RT-PCR, and the differentiation ability of UCB-MSC was examined by alizarine red S staining to confirm the induction level of osteogenic differentiation: (**A**) The expression level of stemness biomarkers and (**B**) the expression level of osteogenic differentiation biomarkers induced by *PKD1* in UCB-MSC; (**C**) The degree of the differentiation into osteocyte in *PKD1*-expressing UCB-MSC via alizarin red S staining. Data shown represent the mean ± SD (*n* = 3). ** *p* < 0.02, *** *p* < 0.01, for UCB-MSC only group versus UCB-MSC/*PKD1* group.

In the results, the expression of all stemness markers in *PKD1*-expressing UCB-MSC was remarkably increased compared with those of UCB-MSC only (Figure 5A). Oct4 and Sox2 increased by about 15 times over UCB-MSC only, respectively. Furthermore, Nanog showed the biggest difference in expression. This result demonstrates that the expression of *PKD1* induces the increased expression of stemness markers in stem cells.

The increased expression of stemness markers will indicate an improvement in the differentiation of stem cells. To explore the differentiation of *PKD1*-expressing stem cell, UCB-MSC was transfected with *PKD1* vector and then replaced with osteocyte differentiation medium when the cell confluence reached 80%. Stem cells were assessed for the degree of differentiation into osteocytes on the 12th day via osteocyte markers, alkaline phosphatase (ALP), and runt-related transcription factor 2 (Runx2) [4,24,25]. Based on the start of differentiation from the 3rd day, it was differentiated until the 12th day, and medium was replaced every three days. As shown in the result of Figure 5B, the expression of ALP and RUNX2 in UCB-MSC treated with *PKD1* vector was relatively increased compared with that of UCB-MSC only, leading to enhanced osteogenic differentiation.

In order to identify these abilities of *PKD1* more visually, *PKD1*-expressing UCB-MSC was stained by alizarin red S solution. Calcium precipitation for the verification of bone cells is not observed in undifferentiated stem cells, but calcium is precipitated in the cells differentiated into bone cells. Therefore, alizarin red S staining was performed to confirm whether the differentiated cells for 12 days were well differentiated into osteocytes. As shown in the result of Figure 5C, *PKD1* transfected UCB-MSC has more of a reddish color compared with that of UCB-MSC, which means that more calcium is precipitated. It was confirmed that the calcium precipitation rate in *PKD1*-transfected UCB-MSC was higher, meaning that *PKD1* improved the differentiation ability.

### 2.4. PKD1 Promotes Osteogenic Differentiation in Tooth Mimetic 3D Model System

**Figure 6:**
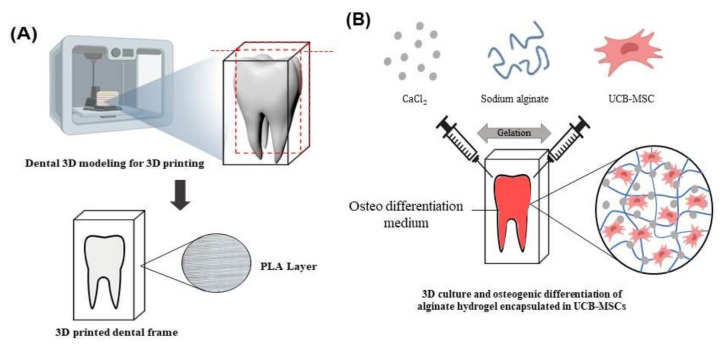

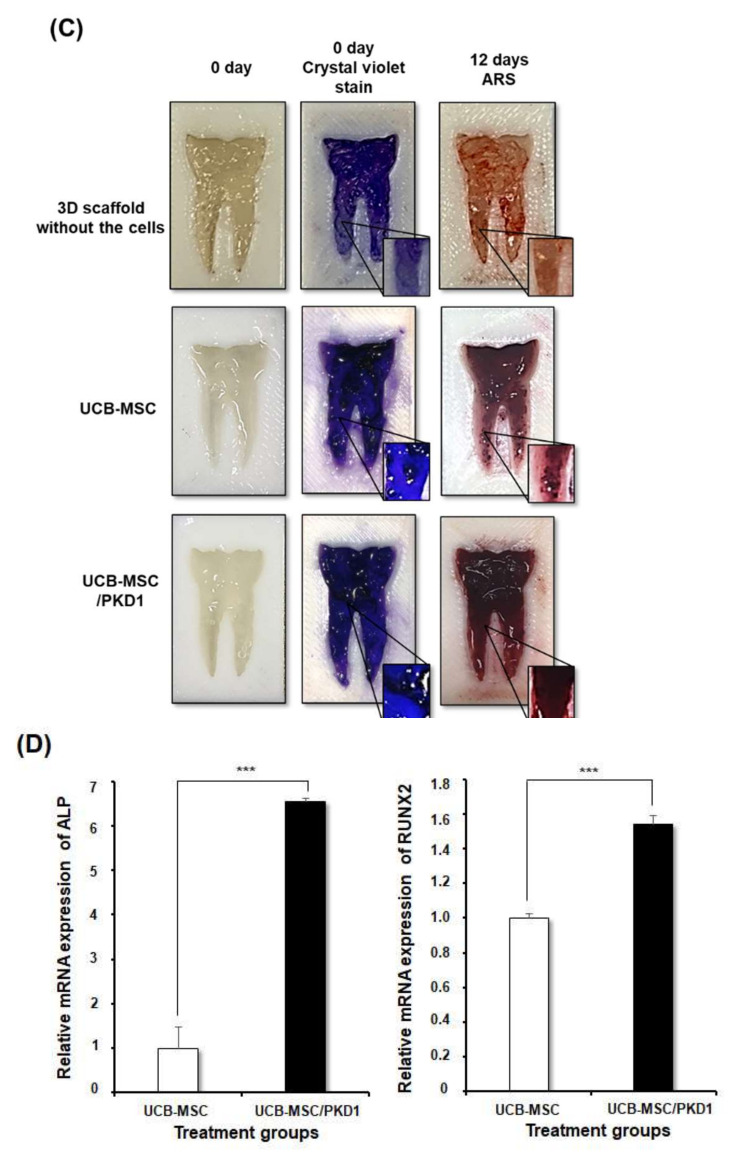
Improved osteogenic differentiation ability of UCB-MSC expressing *PKD1* in a tooth mimetic 3D model by 3D cell culture and 3D printing. (**A**) The fabrication of artificial tooth model by 3D printer technique. (**B**) Tooth mimetic 3D model scheme with alginate hydrogel encapsulated with UCB-MSC/*PKD1* in a 3D tooth template for inducing osteogenic differentiation. (**C**) The images of cell viability and the differentiation levels by crystal violet and alizarin red S staining. (**D**) The mRNA expression levels of osteogenic differentiation markers of UCB-MSC expressing *PKD1* in artificial tooth mimetic 3D model. Data shown represent the mean ± SD (*n* = 3). *** *p* < 0.01 for the UCB-MSC only group versus the UCB-MSC/*PKD1* group.

In the previous experiment, we confirmed that *PKD1* expression induces improved osteogenic differentiation in UCB-MSC. Therefore, we tried to increase the differentiation potency of stem cells by applying a 3D cell culture and tooth mimetic 3D model system to the next. The hydrogel mixture with alginate and UCB-MSC-expressing *PKD1* was added to a tooth model template produced by using 3D printing technology, and the artificial tooth mimetic 3D model system was placed in a culture dish containing differentiation medium and cultured for 12 days to induce steady differentiation. In the result of Figure 6C, when compared with the 3D scaffold without the cells, crystal violet staining to check the existence and cell viability of stem cells encapsulated in the alginate hydrogel showed that the cells were alive well and encapsulated. Alizarin red S staining was used to check the degree of calcium deposition and whether differentiation occurred in the tooth mimic system. As shown in the result, the degree of calcium deposition in UCB-MSC-expressing *PKD1* was observed to be higher than that of scaffold only or UCB-MSC only, based on the strong degree of red color (Figure 6C). Through the experiment result of alizarin red S staining, we checked whether increased osteogenic differentiation was induced by stem cells expressing *PKD1* in a tooth mimetic 3D model system. After the hydrogel dissolution process, RNA extracted from gained stem cells were analyzed by RT-PCR. As shown in the result in Figure 6D, the expression of the increased markers for osteogenic differentiation was observed in UCB-MSC expressing *PKD1* compared with that of UCB-MSC only. The relative expression values of ALP and Runx2 in the UCB-MSC/*PKD1* group were approximately 6.5 and 1.5 times higher than that of UCB-MSC only, respectively. These results mean that stem cells expressing *PKD1* in a tooth mimetic 3D model system have the improved osteogenic differentiation ability in accordance with the previous results, indicating that this system is able to facilitate the clinical application in dental or bone regeneration.

## 3. Discussion

Some studies about *PKD*, which is known as a mutant gene that causes autosomal dominant polycystic kidney disease (*PKD*), reported only basic functions in the kidney. Biologically, the amount of *PKD* protein produced in normal adults is very small, and most are produced in kidney cells before birth. Structurally speaking, this protein is a form that spans the inside and outside of the cell membrane, and it is known that this affects the growth, proliferation, and migration of cells. Among the two types (*PKD1* and *PKD2*) of *PKD* gene, the function of *PKD1* and its role in various cells have not been identified yet. Most recently, some papers have been published on the correlation between *PKD1* and *PKD2* and cancer [7,8,10,17,18]. These studies have revealed many of the functions of *PKD* proteins. The correlation between cancer cell lines and *PKD* was studied through the mTOR and JAK signaling pathway [14,15,16,26]. As a result, unlike what is known, the function of *PKD* showed different results depending on the cancer cell line, and the opposite result was also shown [15]. Therefore, its research is still controversial and limited. Based on these reference papers, we attempted to evaluate the biological function of *PKD1* in stem cells and to overcome the obstacles induced when stem cells are administrated in vivo, using the biological function of *PKD1*.

For this, the expression of the *PKD1* gene in various cells was confirmed, and UCB-MSC showed a low expression level of *PKD1* among stem cells derived from various origins (Figure 1). Therefore, the function of the *PKD1* gene in UCB-MSC was evaluated after the transfection of over-expressing vectors to induce *PKD1* high expression. The confirmation of *PKD1* expression was assessed at the transcript and protein level (Figure 2). In this study, we tried two transfections because stem cells are known to have low transfection efficiency by DNA degradation before going to the nucleus, and they have the properties of primary cells. In addition, considering that the *PKD1* plasmid vector size used in this experiment was 19.5 kbs, the cells were transfected twice. The actual expression of *PKD1* was confirmed through the FACS analysis results, using the characteristic that *PKD1* spans the cell membrane structurally. In conclusion, we checked *PKD1* expression in UCB-MSC despite the large size of *PKD1*.

Based on these results, three kinds of experiments were conducted to evaluate the biological function of *PKD1* in UCB-MSC. The *PKD1* protein is exposed to the outside of the cell membrane and has the function of cell proliferation due to this characteristic. Therefore, in order to confirm whether *PKD1* induces a cell proliferation function in UCB-MSC, the cells transfected with a *PKD1-*overexpressing vector were cultured in a time-dependent manner. As shown in the results of Figure 3A, cell proliferation of *PKD1*-expressing UCB-MSC was decreased compared with the UCB-MSC only group. Unexpectedly, *PKD1* was found to be not helpful for cell proliferation in stem cells, unlike in cancer cells. As aforementioned, *PKD1* controls or regulates the cell proliferation and migration of cells via mTOR and JAK signals. At this point, we think additional experiments with signal pathways are needed as to why *PKD1* does not induce the increased proliferation of stem cells.

It is known that *PKD* affects anti-oxidative effects in cancer for cell survival. This means that *PKD1* improves cell viability by making tissues resistant to changes in the surrounding environment for cancer growth [19,20,21,22]. Therefore, we assessed whether these *PKD1* functions also appear to have resistance in stem cells against oxidative stress. To induce oxidative cellular damage, H_2_O_2_ was treated, and the survival rate of cells was measured. The result in this experiment indicates that *PKD1* did not have resistance to oxidative stress in UCB-MSC (Figure 3B), just as *PKD1* does not affect cell viability and proliferation in UCB-MSC.

Another function of *PKD* is well known to improve cell mobility [27,28]. To address this function in UCB-MSC, migration analysis was performed to evaluate the migration effect of *PKD1*. As a result, the open area at 12 h in *PKD1* expressing UCB-MSC was significantly reduced compared to UCB-MSC only (Figure 4), meaning that *PKD1* regulates stem cell migration.

These results mean that the introduction of these extrinsic substances, which improve the ability of stem cells to move, will facilitate the movement of stem cells to damaged areas and enhance their therapeutic effectiveness. Marco Tatullo et al. reported research results supporting this fact [29]. In this study, they used human platelet lysate (PL) in dental pulp stem cells containing autologous growth factors, instead of FBS. As a result, the PL-added stem cells showed the improved osteogenic, chondrogenic differentiation and migration ability.

Similar to these results, we believe that *PKD1*, which induced expression, will be a useful substance to enhance the function of stem cells. If *PKD1* positively affects the homing effect, which is one of the biggest characteristics of stem cells, it will have potential as a cell therapy for tissue damage with slow cell turnover and low regenerative capacity [30].

In this point of view, we focused on the differentiation of stem cells. One of the many advantages of stem cells for clinical application is their potential ability to differentiate into cells of various tissues. Thus, we tried to examine whether *PKD1* can function in the stemness and differentiation ability of UCB-MSC [23]. First, we evaluated the expression level of three kinds of stemness biomarkers, such as Oct4, Nanog, and Sox2. As it is known, Oct4 is essential for the self-renewal of stem cells, and Oct4 is controlled by SOX2. A decrease in Sox2 accompanies an increase in Oct4 and eventually promotes mesenchymal differentiation. In addition, Nanog is often known to be highly expressed in cancer stem cells, and it eventually functions as oncogenes but increases self-renewal ability in stem cells. As shown in the result of Figure 5A, when the expression of *PKD1* was increased, the expression of stemness markers was significantly increased. It is an important point that the expression of stemness markers is increased. Increasing and regulating the expression of stemness indicators in mesenchymal stem cells using the *PKD1* gene can prolong the lifespan of cells and inhibit aging, and increase cell growth and viability to grow cells so that cells can be cultured at a large scale. This means that there is a possibility to overcome the shortcomings of mesenchymal stem cells and that it has potential for clinical cell therapy and regenerative medicine as well. This result means that *PKD1* can influence differentiation ability by the increased expression of stemness markers in UCB-MSC.

So, next, we assessed whether the increased expression of potential stemness biomarkers by *PKD1* substantially improves the osteogenic differentiation ability of stem cells because UCB-MSC can differentiate into various tissues such as chondrocytes and adipocytes. We induced differentiation into osteocytes [14,31,32]. After transfection, the cells were cultured in an osteocyte differentiation medium for 12 days. At the 12th day, the cells of each group were analyzed by RT-PCR and alizarin red S staining. As a result, when *PKD1* was expressed, representative osteocyte biomarkers Runx2 and ALP were significantly expressed. In addition, as shown in Figure 5C, the visual results using alizarin red S staining solution to evaluate the differentiation potency of *PKD1* demonstrated that *PKD1* can improve the osteogenic differentiation ability of stem cells, leading to an increase in the therapeutic efficacy as cell therapeutics.

To further refine this biological function of *PKD1* in stem cells, we evaluated the differentiation performance of the stem cells expressing *PKD1* by using 3D cell culture and the 3D printing technique. The three-dimension cell culture system is a culture method that artificially creates an environment similar to the shape of cells in the body by reproducing the realistic intracellular microenvironment. When cells are cultured in a 3D environment, the behavior and physiological characteristics of cells show more effectiveness in in vivo culture than in vitro culture. In addition, since cell–cell interaction becomes active and affects cell behavior and intracellular organs, the proliferation, migration, and differentiation of stem cells can be promoted, and 3D cell tissue induces cell aggregation and allows long-term culture [33,34,35,36,37].

To substantially verify the ability for the differentiation induction of *PKD1*, 3D printing technology was used to make an artificial tooth mimetic 3D model applied with a 3D hydrogel cell culture system. The sodium alginate hydrogel used in this experiment is a natural material and has excellent biocompatibility, and when mixed with cells, it can deliver nutrients and oxygen to the inner part of the cells to increase the survival rate of cells [23,25,38].

As shown in the result of Figure 6, UCB-MSCs expressing *PKD1* in a tooth mimetic 3D model showed significant differentiation ability through alizarin red S stain and osteogenic marker expression in the transcriptome. In addition, when comparing the RNA expression levels of ALP and Runx2 in 2D cell culture and 3D cell culture, it can be seen that the expression of Runx2 is higher than that of ALP in a 2D cell culture (Figure 5B and Figure 6D), and ALP expression was higher in a 3D cell culture system. In the process of osteogenic differentiation of UCB-MSC, Runx2, a major transcription factor related to osteoblast differentiation, is first expressed, undergoes the osteoprogenitor process, and then other enzymes including ALP that promote bone formation are involved [39,40,41]. Therefore, the increased ALP expression level of stem cells cultured in a 3D cell culture environment compared with that of UCB-MSC only means that the differentiation is induced faster when *PKD1* is expressed.

The implication of this study is that stem cells expressing *PKD1* have applicable potential for clinical autologous dental treatment that can replace artificial teeth. When a tooth is lost, various side effects are caused by methods to treat this, and the treatment is limited. However, introducing a stem cell-based mimetic tooth cell therapy method as an alternative for this can reduce its side effects. The results of the osteogenic differentiation of stem cells into effective by *PKD1* in our tooth-mimicking 3D model have a future clinically applicable source, and this suggests the possibility of regenerative medicine clinical application using stem cells.

In the end, the *PKD1* gene showed the potential to act as a useful genetic material to overcome the shortcomings of stem cells by strengthening the function in the stem cells.

## 4. Materials and Methods

### 4.1. Cell Culture

The cell lines 293T (human embryonic kidney cell) and A549 (human lung cancer cell) were all purchased from the American Type Culture Collection (ATCC, Manassas, VA, USA). The adipose-derived stem cell (ADSC) and umbilical cord blood-derived mesenchymal stem cell (UCB-MSC) were kindly provided from Dr. Jae-Yoel Cho and Dr. Kyung Sun Kang at Seoul national university in Seoul, Republic of Korea, respectively. 293T and A549 cell lines were cultured in Dulbecco’s Modified Eagle’s Medium (DMEM) (Hyclone, UT, USA) containing 10% fetal bovine serum (FBS), 1% penicillin–streptomycin (PS; Hyclone), and 20% DMEM without PS for ADSC. UCB-MSC were cultured in stem cell conditioned basal medium of KSB-3 (Kangstem Biotech, Seoul, South Korea) and added to KSB-3 Supplements with 10% FBS. Breast cancer-derived stem cell (BCSC) was prepared and cultured by the previous reported method [42]. All cell cultures were maintained at 37°C in a humidified atmosphere containing 5% CO_2_.

### 4.2. Transfection

Each cell line was transfected with *PKD1* overexpressing plasmid in the lentiviral GFP vector (Origene, Rockville, MD, USA) using TransIT-X2 (Mirus Bio, Madison, WI, USA) according to the manufacturer’s instructions. To improve the transfection efficacy and induce high expression of the PKD gene, transfection was repeated twice in the same cells while maintaining minimal toxicity to the cells, and then media was replaced at 24 h post-transfection. Transfected cells were used in experiments after 48 h incubation.

### 4.3. RNA Extraction and Conventional and Quantitative Real-Time Polymerase Chain Reaction (PCR)

At 48 h post-transfection in the experiment, the cells were harvested, and total RNA was extracted according to the manufacturer’s manual using TRIzol reagent (Invitrogen, Carlsbad, CA, USA). Then, it was quantified by Nanodrop^TM^ (Thermo Fisher Scientific, Waltham, MA, USA). The extracted RNA was converted into complementary DNA (cDNA) with DiaStar™ 2× RT Pre-Mix (Solgent, Daejeon, Korea). The synthesized cDNA was subjected to conventional PCR using a Solg™ 2× Taq PCR Pre-Mix (Solgent) according to the manufacturer’s protocols. All samples synthesized by conventional PCR were confirmed by separation through 1% agarose gel electrophoresis (Vivantis, Molecular Biology Grade, CA, USA) in TAE buffer. Gel images were taken using a Chemidoc (VilberLourmat, Eberhardzell, Germany).

Quantitative real-time PCR (qPCR) was conducted in a CFX96^TM^ real-time system employing Solg™ 2× Real-Time PCR Smart mix (including SYBR Green in the mixture, Solgent). The primer sequences used in this study are presented in Table 1.

### 4.4. Flow Cytometry Analysis

After preparing the sample, *PKD1* monoclonal antibody (sc-130554, Sant Sigma, UK; a Cruz Biotechnology, Santa Cruz, CA, USA), 5 μg/mL final concentration, is added to each of at least 1.5 × 10^5^ cells per 100 μL and incubated at 4 °C for 2 h. After the reaction time between the cells and the *PKD1* antibody, the cells were washed out with 500 μL of DPBS, and then a secondary antibody was added (4 μg/mL final concentration, Alexa Fluor 488-conjugated goat anti-mouse antibody; Thermo scientific, Waltham, MA, USA) to 100 μL of DPBS and reacted at 4 °C for 30 min. After second antibody staining, the cells were analyzed using a flow cytometer (NovoCyte, ACEA Biosciences Inc., San Diego, CA, USA).

### 4.5. Cell Viability Assay under Oxidative Condition

Each cells transfected with lentivirus-expressing *PKD1* gene and positive control GFP-expressing gWIZ/GFP vector were treated with 5 mM of hydrogen peroxide solution (H_2_O_2_) for 4 h and then recovered with complete media for 1 day. The cell viability effect of the *PKD1* gene against oxidative stress was assessed by 3-(4,5-dimethylthiazol-2-yl)-2,5-diphenyltetrazolium bromide (MTT) (Sigma-Aldrich, Poole, UK) After removing the cell culture medium in each well, MTT reagent was added and reacted for 4 h at 37 °C in a CO_2_ incubator. After the reaction, the supernatant was removed, and then dimethyl sulfoxide (DMSO; Sigma-Aldrich, St. Louise, MO, USA) was added to dissolve the formazan crystals formed in the cells, and the absorbance was read at 570 nm by a microplate reader (Versamax microplate reader; Molecular Devices Corp., Sunnyvale, CA, USA) instrument. Cell viability was expressed as a percentage using the absorbance value for the control. The non-treated cell group was analyzed as a negative control. Each data point represents the mean of the triplicate experiment.

### 4.6. Cell Proliferation Assay

Cells were seeded onto 48-well plates at 2 × 10^4^ cells per well. The *PKD1* gene was transfected into UCB-MSC, as described above, according to each condition. After giving a recovery time of 24 h in a 37 °C incubator, cell viability was measured by MTT assay depending on time from 24 to 72 h to confirm cell proliferation [15].

### 4.7. Cell Migration Assay

Stem cells were seeded onto 24-well plates at 8 × 10^4^ cells per well. When the confluence of transfected cells prepared in each group reached 80%, they were scraped to make it uniformly wide at regular intervals with a 200 μL sterile pipette tip [26,27,31]. The photographs were taken at 0 and 12 h after scratch creation with a microscope (Nikon eclipse Ts2R, Nikon, Tokyo, Japan) [28].

The 12 h sample in each group was stained with crystal violet dye to check for visible differences. After dissolving the crystal violet solution in methanol to a concentration of 0.1%, 1 mL of 0.1% crystal violet dye was added to the well. When cell fixation and staining are complete, the wells were carefully washed out 5 times with sterilized water and dried at room temperature before taking a picture. The ratio of cell movement in the 12 h sample compared to the 0 h sample was quantitatively analyzed by the software called image J. [23,43,44,45,46].

### 4.8. The Induction of Osteogenic Differentiation by PKD1 Expression

In order to evaluate the differentiation ability of stem cells according to *PKD1* expression level, the medium of *PKD1* transfected UCB-MSCs was replaced with osteogenic differentiation medium (Stem-pro osteogenesis differentiation kit, Invitrogen, Gibco) [4,14,26,47]. It was replaced with a new medium every three days, and we confirmed the degree of differentiation to the osteogenic cells via alizarin red S staining and osteoblast biomarkers, such as runt-related transcription factor 2 (Runx2) and Alkaline phosphatase (ALP), on the 12th day [31]. To visually identify the degree of differentiation into osteogenic cells, UCB-MSC was treated with alizarin red S staining solution to observe the degree of calcium salt deposition in the cells following the differentiation of UCB-MSC into osteocyte. Alizarin red S mono sodium salt (Merck, Darmstadt, Germany) was prepared and used according to the manufacturer’s manual [47,48]. The photograph was taken with a microscope (Nikon eclipse Ts2R, Nikon, Tokyo, Japan).

### 4.9. The Manufacture of Tooth Model Using 3D Printer

To evaluate the osteogenic differentiation ability of UCS-MSC expressing *PKD1* in ex vivo condition, a tooth model using a (3D) printer was generated. To manufacture the tooth template by 3D printer, poly lactic acid (PLA) filament was used and a Formlabs form2 printer (Formlabs, Somerville, MA, USA) using stereolithography (SLA) method was used, and SLA has the advantage of producing a robust model with a thin layer because it can predict the output result. A tooth picture was created and modeled using PreForm 3D printing software based on a model that mimics a real tooth. We printed at a speed of 40 mm/s at a 3D printer nozzle temperature of 210 degrees and a bed temperature of 60 degrees, and the thickness of the print layer was set to 0.1 mm. Since the SLA printing method requires a post-processing process, the build plate of the printed model was removed and dipped in a finishing kit. Finally, after drying, the surrounding support is removed and used in the experiment.

### 4.10. D Cell Culture System by Sodium Alginate Hydrogel

In order to grow UCB-MSC expressing *PKD1* to 3D cell culture system in a 3D tooth model, hydrogel was manufactured using alginate, which is a natural polymer material in a teeth template. Sodium alginate and calcium chloride (CaCl_2_) prepared in a 3 mL syringe were reacted with each other to form a hydrogel via gelation. At this time, to generate hydrogel encapsulated with UCB-MSC/*PKD1*, stem cells (2 × 10^4^ cells/mL) were suspended in the 2% alginate solution and transferred to a syringe and then were co- mixed with 200 mM CaCl_2_ solution. The mixture hydrogels were incubated in osteogenic differentiation medium for 12 days. Differentiated cells were identified by Alizarin red S staining and RT-PCR to confirm osteogenic differentiation levels.

### 4.11. Statistical Analysis

All experiments were performed at least three times repetitive experiments, and the measured data were calculated as mean ± standard error of the mean (SEM) [19] and presented as a graph. The significance test between groups was analyzed by independent sample *t*-test and one-way ANOVA. Statistical differences were indicated in the figures. * p < 0.05, ** p < 0.02, *** p < 0.01. For statistical analysis, SPSS statistics software for Windows, Version 18 (SPSS Inc., Chicago, IL, USA) was used.

## 5. Conclusions

This study is the first report to evaluate the biological function of the *PKD1* gene in UCB-MSC. Stem cells with potential capabilities are now widely used in medical treatment. However, there are limitations to stem cell therapy. It is difficult to cultivate stem cells, and useful genetic materials were researched to compensate for various side effects and disadvantages in the restriction of the number of subcultures and differentiation. Here, we focused on the *PKD1* gene. When the *PKD1* gene was applied to UCB-MSC, the cell migration ability of stem cell was improved, and enhanced stemness induced the osteocyte differentiation of stem cells in a tooth mimetic 3D model by the 3D printing technique. Taken together, our study ascertains that *PKD* has the potential as another option that can complement the shortcomings of stem cells and enhance differentiation.

## Figures and Tables

**Table 1:** Primer sequences used for PCR analysis.

Gene	Accession No.	Direction	Primer Sequence (5′ to 3′)
*GAPDH*	NM_002046	Forward	5′-AGG GCT GCT TTT AAC TCT GGT-3′
		Reverse	5′-CCC CAC TTG ATT TTG GAG GGA-3′
*PKD1*	NM_001009944.3	Forward	5′-ATG ACT GGC TTT CGG TGG AG-3′
		Reverse	5′-GGA GGC CTG AGA ACG TGA G-3′
*Oct4*	NM_112957.3	Forward	5′-CCT GAA GCA GAA GAG GAT CAC C-3′
		Reverse	5′-AAA GCG GCA GAT GGT CGT TTG G-3′
*Nanog*	NM_024865.4	Forward	5′-CTC CAA CAT CCT GAA CCT CAG C-3′
		Reverse	5′-CGT CAC ACC ATT GCT ATT CTT CG-3′
*Sox2*	NG_009080.1	Forward	5′-GCT ACA GCA TGA TGC AGG ACC A-3′
		Reverse	5′-TCT GCG AGC TGG TCA TGG AGT T-3′
*ALP*	NM_001369804	Forward	5′-TGG AGC TTC AGA AGC TCA ACA CCA-3′
		Reverse	5′-ATC TCG TTG TCT GAG TAC CAG TCC-3′
*Runx2*	NM_001024630.4	Forward	5′-TTA CCC CTC CTA CCT GAG CC-3′
		Reverse	5′-TTC CAT CAG CGT CAA CAC CA-3′

## Supplementary Materials

The following are available online at https://www.mdpi.com/article/10.3390/ijms22094868/s1. Figure S1: Verification of *PKD1* expressing lentiviral vector. Table S1: The measured space values of the cells migrated by *PKD1* expressing UCB-MSC.
