# Supplementary material for: Polycystin-1 Enhances Stemmness Potential of Umbilical Cord Blood-Derived Mesenchymal Stem Cells
Source: Int J Mol Sci. 2021 May 4;22(9):4868. doi: 10.3390/ijms22094868 (PMC8125233; doi:10.3390/ijms22094868)
Supplement: Supplementary file 1 [file ijms-22-04868-s001.zip › ijms-1168814-supplementary.pptx]

## Slide 1
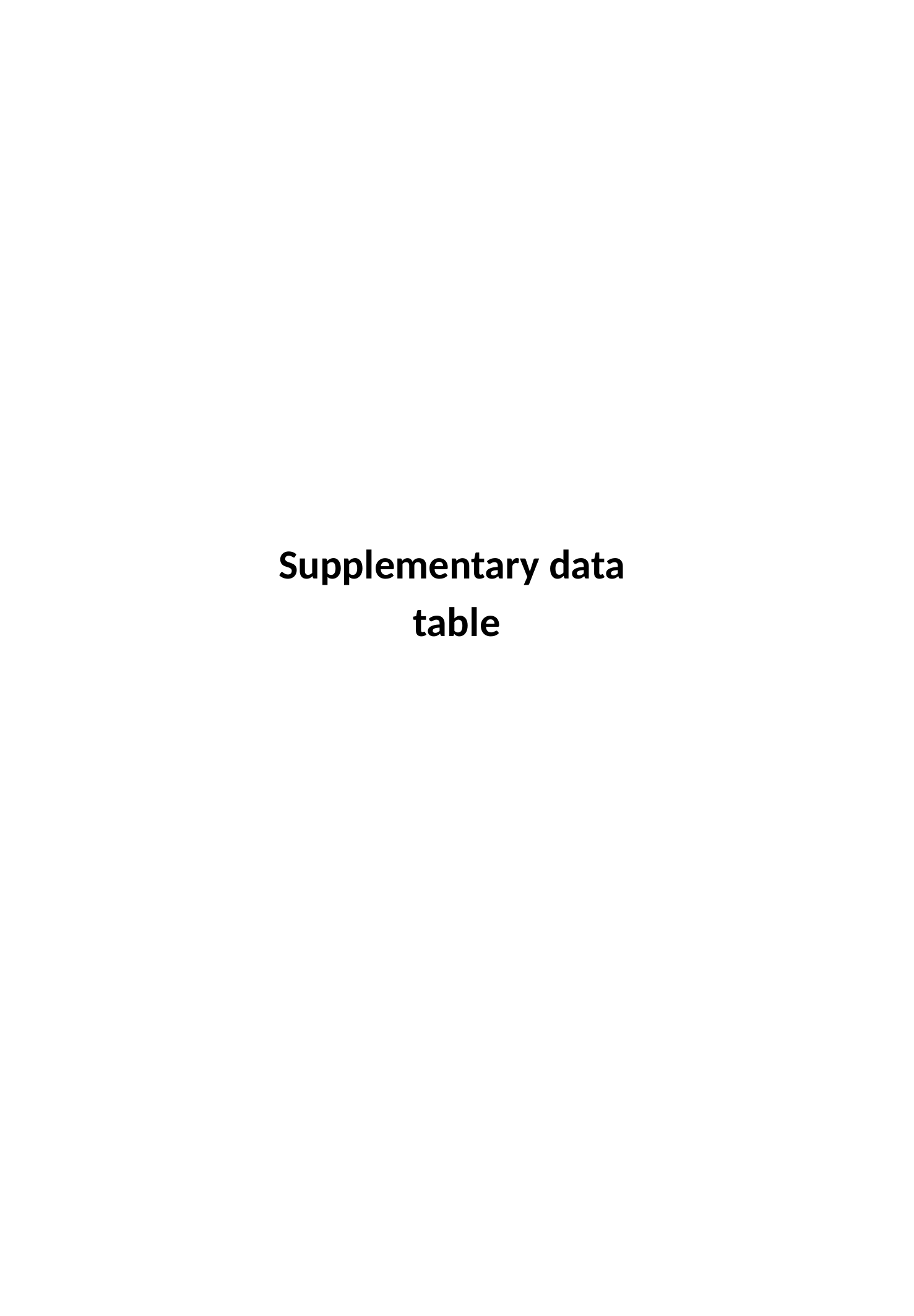

Supplementary data
table

## Slide 2
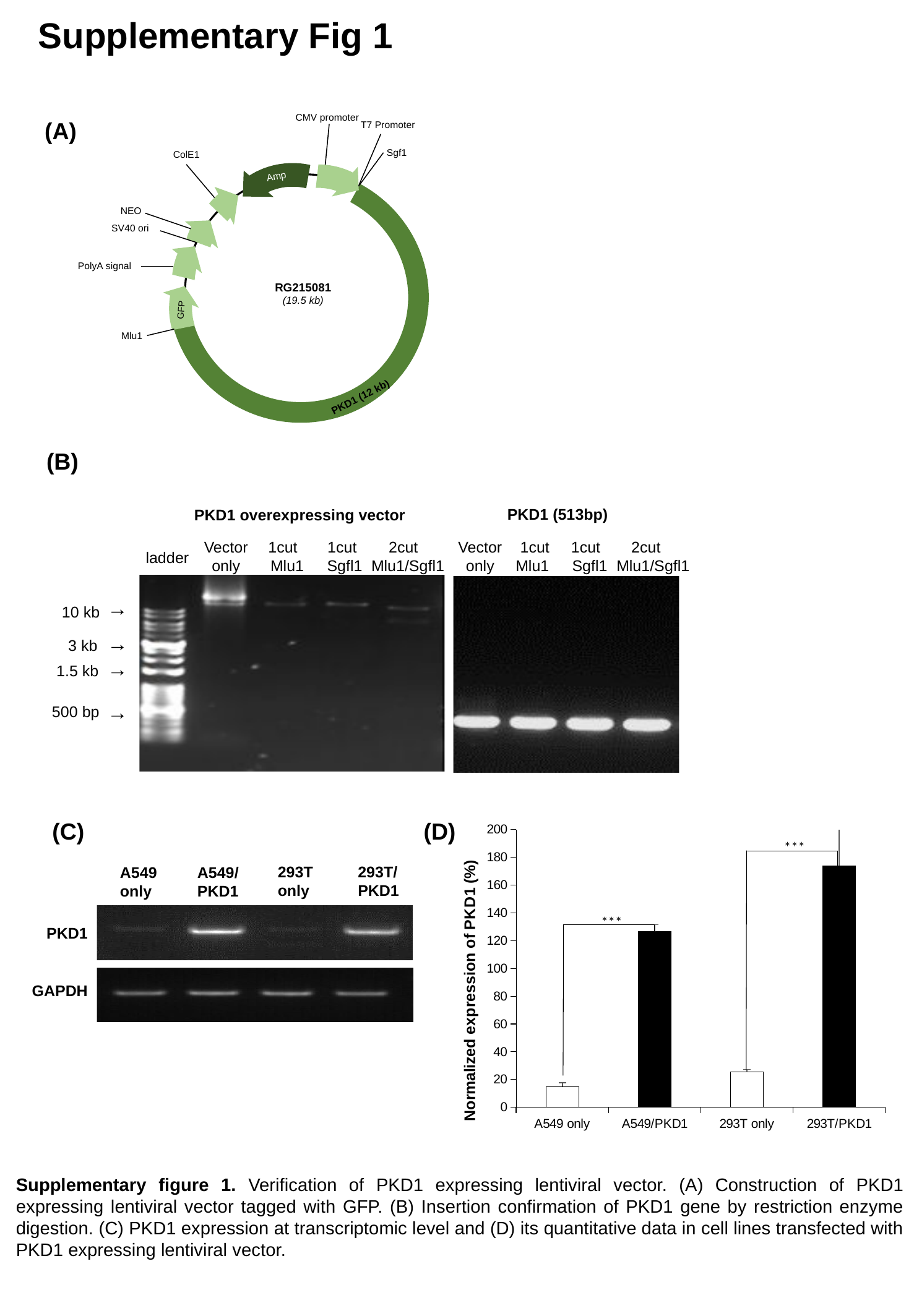

Supplementary Fig 1
CMV promoter
T7 Promoter
Sgf1
ColE1
Amp
NEO
SV40 ori
PolyA signal
RG215081
(19.5 kb)
GFP
Mlu1
PKD1 (12 kb)
(A)
(B)
PKD1 (513bp)
PKD1 overexpressing vector
 1cut
 1cut
 1cut
 2cut
 1cut
 2cut
Vector
only
Vector
only
ladder
Mlu1
Sgfl1
Mlu1/Sgfl1
Mlu1
Sgfl1
Mlu1/Sgfl1
→
10 kb
→
3 kb
→
1.5 kb
→
500 bp
### Chart
| Category | |
|---|---|
| A549 only | 14.99994102544526 |
| A549/PKD1 | 126.44968652610406 |
| 293T only | 25.405447256626296 |
| 293T/PKD1 | 174.09208298729305 |(C)
(D)
***
293T
only
293T/
PKD1
A549
only
A549/
PKD1
***
PKD1
GAPDH
Supplementary figure 1. Verification of PKD1 expressing lentiviral vector. (A) Construction of PKD1 expressing lentiviral vector tagged with GFP. (B) Insertion confirmation of PKD1 gene by restriction enzyme digestion. (C) PKD1 expression at transcriptomic level and (D) its quantitative data in cell lines transfected with PKD1 expressing lentiviral vector.

## Slide 3
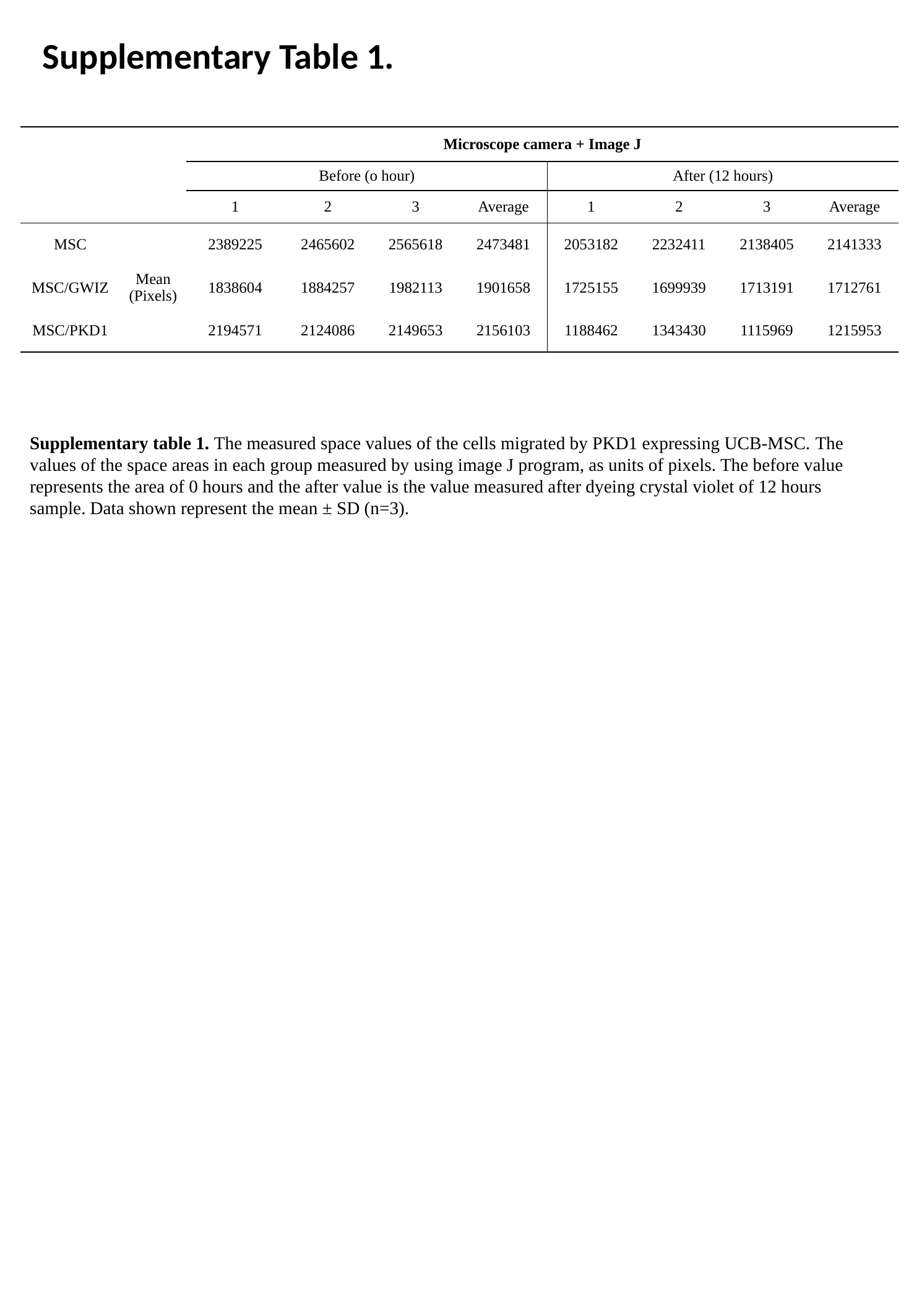

Supplementary Table 1.
| | | Microscope camera + Image J | | | | | | | |
| --- | --- | --- | --- | --- | --- | --- | --- | --- | --- |
| | | Before (o hour) | | | | After (12 hours) | | | |
| | | 1 | 2 | 3 | Average | 1 | 2 | 3 | Average |
| MSC | Mean (Pixels) | 2389225 | 2465602 | 2565618 | 2473481 | 2053182 | 2232411 | 2138405 | 2141333 |
| MSC/GWIZ | | 1838604 | 1884257 | 1982113 | 1901658 | 1725155 | 1699939 | 1713191 | 1712761 |
| MSC/PKD1 | | 2194571 | 2124086 | 2149653 | 2156103 | 1188462 | 1343430 | 1115969 | 1215953 |
Supplementary table 1. The measured space values of the cells migrated by PKD1 expressing UCB-MSC. The values of the space areas in each group measured by using image J program, as units of pixels. The before value represents the area of 0 hours and the after value is the value measured after dyeing crystal violet of 12 hours sample. Data shown represent the mean ± SD (n=3).
